# Mechanisms of Basin-Scale Nitrogen Load Reductions under Intensified Irrigated Agriculture

**DOI:** 10.1371/journal.pone.0120015

**Published:** 2015-03-19

**Authors:** Rebecka Törnqvist, Jerker Jarsjö, Josefin Thorslund, P. Suresh C. Rao, Nandita B. Basu, Georgia Destouni

**Affiliations:** 1 Department of Physical Geography and Quaternary Geology and the Bolin Centre for Climate Research, Stockholm University, Stockholm, Sweden; 2 School of Civil Engineering and Department of Agronomy, Purdue University, West Lafayette, West Lafayette, Indiana, United States of America; 3 Department of Civil and Environmental Engineering and Earth and Environmental Sciences, University of Waterloo, Waterloo, Ontario, Canada; St. Francis Xavier University, CANADA

## Abstract

Irrigated agriculture can modify the cycling and transport of nitrogen (N), due to associated water diversions, water losses, and changes in transport flow-paths. We investigate dominant processes behind observed long-term changes in dissolved inorganic nitrogen (DIN) concentrations and loads of the extensive (465,000 km2) semi-arid Amu Darya River basin (ADRB) in Central Asia. We specifically considered a 40-year period (1960–2000) of large irrigation expansion, reduced river water flows, increased fertilizer application and net increase of N input into the soil-water system. Results showed that observed decreases in riverine DIN concentration near the Aral Sea outlet of ADRB primarily were due to increased recirculation of irrigation water, which extends the flow-path lengths and enhances N attenuation. The observed DIN concentrations matched a developed analytical relation between concentration attenuation and recirculation ratio, showing that a fourfold increase in basin-scale recirculation can increase DIN attenuation from 85 to 99%. Such effects have previously only been observed at small scales, in laboratory experiments and at individual agricultural plots. These results imply that increased recirculation can have contributed to observed increases in N attenuation in agriculturally dominated drainage basins in different parts of the world. Additionally, it can be important for basin scale attenuation of other pollutants, including phosphorous, metals and organic matter. A six-fold lower DIN export from ADRB during the period 1981–2000, compared to the period 1960–1980, was due to the combined result of drastic river flow reduction of almost 70%, and decreased DIN concentrations at the basin outlet. Several arid and semi-arid regions around the world are projected to undergo similar reductions in discharge as the ADRB due to climate change and agricultural intensification, and may therefore undergo comparable shifts in DIN export as shown here for the ADRB. For example, projected future increases of irrigation water withdrawals between 2005 and 2050 may decrease the DIN export from arid world regions by 40%.

## Introduction

The global agriculture production system has heavily altered and accelerated the nitrogen (N) cycle by introducing synthetic N fertilizers, in addition to animal manure, to produce food for a growing world population, see e.g., [1–4]. The global average of N use efficiency in agriculture is around 50% [5]. However, it is spatially variable and can for instance be lower than 20% in Asian rice fields and be above 60% in Europe and North America [5–6]. This implies that large amounts of the applied N are lost to the groundwater and surface water systems. Such N leaching impairs water quality and contributes to eutrophication in lakes and coastal systems, see e.g., [7–9]. A considerable increase in the export of anthropogenic N through the world’s rivers has also been observed [10].

Improvement of agricultural practices can decrease N loads through more precise fertilizer application techniques that reduce such losses and the need for excess fertilizer use. For instance, decreased fertilizer use in many European countries since the mid-1980s have caused N river loads to be constant or decrease in several European rivers [11–12]. N concentrations and loads can also be naturally attenuated during their way in the soil-water system. N attenuation processes can be defined as physical, chemical, and biological processes that reduce the mass, mobility, and/or concentration of N in soil-water systems [13]. This definition of N attenuation thus includes N retention in the system. Agricultural practices can enhance N attenuation processes such as by creating conditions favorable for denitrification in drainage channels and irrigated top soil [14–16]. N attenuation in agricultural systems generally occurs by delay of N transport through storage in soil, groundwater, perennial vegetation etc., and irreversibly by crop harvest and losses of N to the atmosphere through denitrification processes [16].

In many parts of the world, and especially in arid and semi-arid regions, agricultural development is also associated with irrigation expansion. The large water diversions and water losses that are often associated with irrigation can considerably modify the cycling of water, nutrients and other elements [16–17]. Combined effects of changed water flows and nutrient releases associated with agricultural intensification are difficult to quantify on catchment scales due to incomplete knowledge of the processes involved and their variable impacts in space and time. However, flow-path modification through recycling of drainage water to irrigated fields has been shown to decrease mass flows and concentrations of N at the plot scale, for instance by making conditions more favorable for denitrification in flood irrigated paddy fields, see, e.g., [14, 18–19]. Arid river basins have been found to be characterised by higher N attenuation compared to mesic river basins, mainly due to a higher degree of denitrification losses of N and larger density of reservoirs which increase the water residence time [20]. Furthermore, accumulation of N legacies in the subsurface part of managed hydrological catchments can cause more temporally homogenous N loading due to buffering of temporal variation in N concentrations [21].

Projected continued agricultural intensification, net expansion of irrigation and increased aridity in some of the water-scarce parts of the world [22–23], necessitates a more efficient use of water resources. For predicting future trends of N cycling in such regions, and in order to take measures to mitigate possible local or downstream adverse effects, understanding of processes that control nutrient loading from entire river basins and other surface waters (lake or sea) catchments is essential. The aim of this study is to identify dominant processes that influence N loading and cycling in a highly managed and irrigated drainage basin. We specifically consider the Amu Darya River Basin (ADRB), which is the largest sub-basin of the Aral Sea Drainage Basin (ASDB), covering 1.3% of the earth’s land surface. In the ASDB, the net vapor flux to the atmosphere has considerably increased since the 1960s, mainly due to a vast irrigation expansion that has in turn led to a drastic decrease in river discharge [17] and associated well-known drying of the Aral Sea, along with an overall freshwater scarcity in the basin [24]. In terms of scale and magnitude, the hydrological consequences of the agricultural expansion within ASDB are unprecedented. Therefore, a main working hypothesis is that analysis of data on ASDB’s clearly changing hydrology, land use and contamination since the 1960s may reveal long-term trends and underlying causal factors that (as of yet) may be masked by random fluctuations in many other catchments. Specifically, we investigate changes in N concentrations and loads near the Aral Sea outlet during the period 1960–2000 and aim to relate this to land-water use changes such as changed N fertilizer application, irrigated area, discharge and diversions of irrigation water.

## Data and Methods

### 2.1 Site description

**Fig 1:**
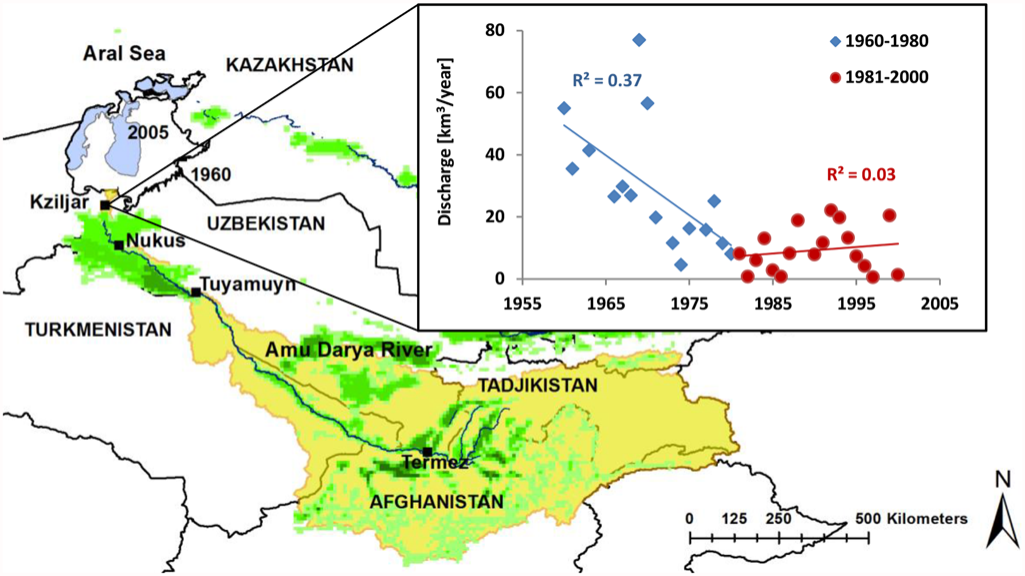
The Amu Darya drainage basin (outlined in yellow) with the Amu Darya River and gauging station Kziljar located in the Amu Darya River delta south of the sea, and the upstream gauging stations Nukus, Tuyamuyn reservoir complex and Termez. The extent of the Aral Sea in 1960 (white) and in 2005 (filled blue), irrigated areas (green), and temporal trend in annual mean discharge at Kziljar station for the periods 1960–1980 (blue), and 1981–2000 (red) are shown.

The ADRB lies within the borders of Kyrgyzstan, Tajikistan, Turkmenistan, Uzbekistan and Afghanistan and has an extent of about 465,000 km^2^ [25], which approximately corresponds to the area of Spain (see yellow area in Fig. 1). Two-thirds of the basin’s irrigated areas (green in Fig. 1) are located in Uzbekistan. The main part of the discharge (about 85%) of the 2550 km long Amu Darya River originates from precipitation (P) in the glaciated Pamir, Tienshan and Hindukush mountain range in the south-east part of the river basin [26–27]. The western part of the river basin is low laying land with about 96% of the area receiving P of less than 300 mm/year [28]. Coarse to medium Quaternary sediment soils prevail in the mountainous part of the basin, and Quaternary and Pliocene alluvial sand, loam, and clay soils prevail in the arid plain part of the basin [28]. The soil organic N levels have been reported to range between 0.012–0.073% [29]. Large amounts of fertilizers are used (of up to 250 kg N/ha), in particular for cotton and wheat [30]. N concentrations above health-risk based drinking water guideline values from the World Health Organization of 11 mg/l as NO_3_-N and 0.9 mg/l as NO_2_-N [31] have occasionally been observed in surface waters in downstream regions of the ADRB [32].

The irrigated areas in the ADRB started to expand in the beginning of the 20^th^ century. However, the expansion did not intensify until in the middle of the century, when cotton monoculture greatly increased in the region (Table 1; S1a Fig.) [33]. Today, about one third of the irrigated areas in the drainage basin are cultivated with cotton, and wheat production is becoming increasingly important for reaching self-sufficiency of grains [34–35]. River flows are regulated by upstream and downstream reservoirs [36]. Surface irrigation, and in particular furrow irrigation in which irrigation water is led in trenches between crop rows, is the dominant irrigation practice and the irrigated soils are continuously leached to keep the salinized soils’ salinity at an acceptable level [37]. Return flows from irrigated fields are considerable in the catchment with its complex irrigation-drainage channel system [38].

**Table 1:** Changes in area irrigated [39–40], irrigation water diversion [39], irrigation water use per hectare, N fertilizer application rates per hectare, data from FAO’s statistical database FAOSTAT (available at faostat.fao.org) and [35], and total N fertilizer application between the years of 1960, 1980 and 2000 in the Amu Darya river basin (only the former Soviet Central Asia is considered).

Year	Area irrigated [ha]	Irrigation water diversion [km^3^/year]	Irrigation water use per hectare [m^3^/ha]	N fertilizer application rate in ADRB [kgN/ha]	Total N fertilizer application in ADRB [ton/year]
**1960**	2,045,000	32	15,600	61	160,000
**1980**	2,791,000	56	20,000	120	405,000
**2000**	3,650,000	47	12,900	99	419,000

Field studies from Uzbekistan have shown that the N recovery of the N applied as fertilizer is only about 35% for cotton and wheat crops [29]. The major part of the applied N fertilizers is thus either lost by leaching and denitrification, up to 70% [29; 41–42] or immobilized in the soil, up to 50% [29–30]. Denitrification rates in the irrigated fields of the ADRB are reported to be high due to favorable conditions of denitrification through coupled irrigation and fertilization events [41;43].

### 2.2 Data sources and synthesis

We used monthly data on discharge and concentrations of nitrate (NO_3_^-^) and nitrite (NO_2_^-^) for the period 1960–2000, and monthly data on concentrations of ammonium (NH_4_^+^) for the period 1977–2000, measured in the Amu Darya River near its Aral Sea outlet at Kziljar (see Fig. 1). Dissolved inorganic N (DIN) concentrations were computed as the sum of NO_3_-N, NO_2_-N and NH_4_-N concentrations. We here focus on DIN since it comprises highly soluble and dominant (especially NO_3_^-^) forms of nitrogen in agriculturally impacted river systems, e.g. [44–45], and since monitoring data existed for these fractions. Although not monitored, we recognize that dissolved organic nitrogen (DON) and particulate N exist in the basin. However, literature shows that they are less important for total mass balances than DIN, since ammonification and nitrification processes will largely convert these forms into soluble NO_3_^-^, e.g., [45–47]. For instance, Vanni et al. [47] estimated particulate N to account for only 5–9% of total N flux in three agricultural basins in the US. Kessel et al. [45] showed that although DON can account for up to a considerable 26% of total soluble N, processes occurring at the source zone convert most of the DON into DIN, making the latter parameter the more relevant one to measure in transport quantifications.

Measurement data were on average available for 7 months per year. Except for lacking measurements during December and January, all months were well represented in the data series. For analysis of long-term changes, data were divided into two equally long periods, 1960–1980 (the early period; measurements for 16 years), and 1981–2000 (the late period; measurements for 18 years). This division implies that each period had sufficient number of data points to reduce potential errors related to random inter-annual fluctuations in weather or other ambient conditions. Furthermore, change-point analysis [48] of the full DIN concentration dataset suggests that a shift in concentration trends occurred in 1981. The two chosen periods therefore also represent the conditions that prevailed before and after that shift. In addition to the Kziljar station, concentration data was also synthesized for three upstream stations (Termez, Tuyamuyn and Nukus, see Fig. 1).

The data were compiled from the Uzbek Centre of Hydro-meteorological service (Uzhydromet) annals and have previously been used in, e.g., [49]. The data were sampled and analyzed with standard methods by the Soviet Union Unified Federal Service for Observation and Control of Environmental Pollution (OGSNK) until 1991 and by the Uzbek Centre of Hydrometeorological service (Uzhydromet) after 1991. For NO_3_^-^ and NO_2_^-^, the standard analysis involved photometric detection with Griess reagent both in the Former Soviet Union [50] and in Uzbekistan [32], implying that the main NO_3_^-^and NO2^-^ analysis method remained unchanged during the here considered period (1960–2000). Furthermore, a comparison of the here used NO_3_^-^and NO2^-^ concentration data (for 1998–2000) with parallel data obtained through current international standard analysis methods [51], showed similar results [32].

For NH_4_^+^, concentration measurements were lacking during the period 1960 to 1976, and specific (annual) NH_4_^+^ contributions to DIN could therefore not be calculated for this period. Furthermore, the Soviet Union standard reagent for photometric detection of NH_4_^+^ changed around 1979 [50], from Nessler that reportedly yielded elevated analysis values of NH_4_^+^ [52–53] to indophenol blue that subsequently (after 1991) was adopted as Uzbek standard [32]. The potential impacts of this early-period NH_4_^+^ data gap and the subsequent change in NH_4_^+^ analysis method were estimated by considering NH_4_^+^ concentration measurement results and comparing it to concentrations of the other DIN constituents (NO_3_^-^and NO_2_^-^). Specifically, we estimated how much lacking NH_4_^+^ measurements and method-related overestimations of NH_4_^+^ could possibly contribute to estimated average DIN concentrations of considered periods 1960–1980 and 1981–2000 (and related concentration difference between the periods).

Annual loads of DIN were calculated as the annual mean concentration times the annual mean water discharge. Annual loads were also estimated from a processed monthly data series as the sum of monthly average concentration times the monthly average discharge. In the processed data series, months that lacked original data were filled by concentration estimates. This was done by increasing the total concentration and discharge during missing months according to the historical relative contribution of that specific month to the annual concentration or discharge. The historical relative contribution of each month was calculated empirically from a 25-year monthly concentration and discharge pattern of the upstream station Nukus (see Fig. 1 for location), and scaling it to the concentration level of the months measured. Results from this alternative load and concentration calculation were compared to results from the annual average approach of available data. Annual flow-weighted mean concentrations were calculated by dividing the sum of monthly load by the sum of monthly water discharge. Annual trend analyses of discharge, DIN concentration and DIN load was performed. Linear correlation between annual DIN load and discharge, DIN load and concentration as well as between annual DIN load and DIN concentration was performed for 1960–2000, 1960–1980 and 1981–2000. It should be noted that both discharge and concentration are auto-correlated to load. However, we wanted to investigate if the DIN load was predominantly dependent on discharges or concentrations. The slope of lines fitted to the load-discharge relations for the two periods 1960–1980 and 1981–2000 was tested for statistically significant differences using the Welch test, which is a two-sample t-test for unequal variances. The coefficient of variation (CV) was further calculated for discharge, DIN concentration and DIN load for the two periods 1960–1980 and 1981–2000. Ratios of observed concentrations between 1960–1980 and 1981–2000 periods were calculated for Kziljar and the three other considered stations along the Amu Darya River (i.e., Termez, Tuyamuyn and Nukus see Fig. 1).

Data on irrigated area per country were taken from FAO Aquastat [40]. The Global Map of Irrigation [54] was used to determine each country’s percent irrigated area within the ADRB. N fertilizer application rate per country in 2000 was taken from FAO’s statistical database FAOSTAT (available at faostat.fao.org). The N fertilizer application rate for the whole basin was calculated based on the percentage of a country’s irrigated area in the basin assuming a relatively uniform application across the whole country. For instance, for Uzbekistan, which uses about 70% of the total N fertilization in the basin, data on regional N fertilizer application per hectare [55] showed that relatively uniform fertilizer application was used across the whole country (7% difference in application rate between the part of the country inside and outside the ADRB). Percentage changes in N fertilization (in kgN/ha/year) for the period 1960–2000 in Uzbekistan [29], were assumed to equal the changes in the whole basin. The N crop uptake per hectare was calculated based on the respective percentage of cultivated area of cotton and wheat for the two time periods [39] and measured N uptake per hectare for cotton and wheat [29]. N content in crops (kgN/ton) was calculated as N uptake per hectare divided by crop yield per hectare [29]. Net N input (NNI) was calculated as total N fertilizer application in tons per year minus total N uptake by crops in tons per year. Only the Uzbek part of the ADRB (UADRB) was considered when calculating N crop uptake, N content in crop yield and NNI owing to lack of available data for the other countries. To evaluate the potential influence of dam constructions on DIN concentrations and local attenuation, observed concentrations along the Amu Darya River were compared between the early period (1960–1980) and the late period (1981–2000) during which several dams were taken into operation (Fig. 1).

### 2.3 Quantification methods and models

An analytical relationship for N attenuation under conditions of intensified irrigation and recirculation of irrigation water was developed and compared to observed changes. Distributed, annual average water flows of the ASDB and ADRB were quantified using the water module of the PCRaster-based Polflow model [56], adopted to the ASDB as detailed in [17; 38;57]. Spatially distributed annual averaged temperature (T) and P data for the considered period (1960–2000) from the Climate Research Unit TS 2.1 database, with a resolution of 0.5°, [58] were used as input to the model. ET (including both evaporation and transpiration) was calculated according to the [59] and [60] relations, following the steps of [38]. Specifically, two model runs were made per period, one without water rerouting, yielding ET under natural (pre-irrigation) conditions (ET_nat_), and one with water rerouting representative of the period, yielding a total ET of both rainwater and irrigation water (ET_tot_). The recirculated water flows (Q_r_) for the periods 1960–1980 and 1981–2000 were obtained from corresponding average country-specific irrigation water withdrawals reported in Aquastat [40]. Modeled as a river water sink term, Q_r_ is transferred to and spread over the model’s irrigated fields, spatially delineated according to the Global Map of irrigation areas considering the percentage of irrigation per grid-cell)[54], as extra P, i.e., P_irr_ = Q_r_. The increased ET that can be associated with irrigation (ET_irr_) was determined as ET_tot_-ET_nat_, obtained from the model runs with (ET_tot_) and without (ET_nat_) water routing.

**Fig 2:**
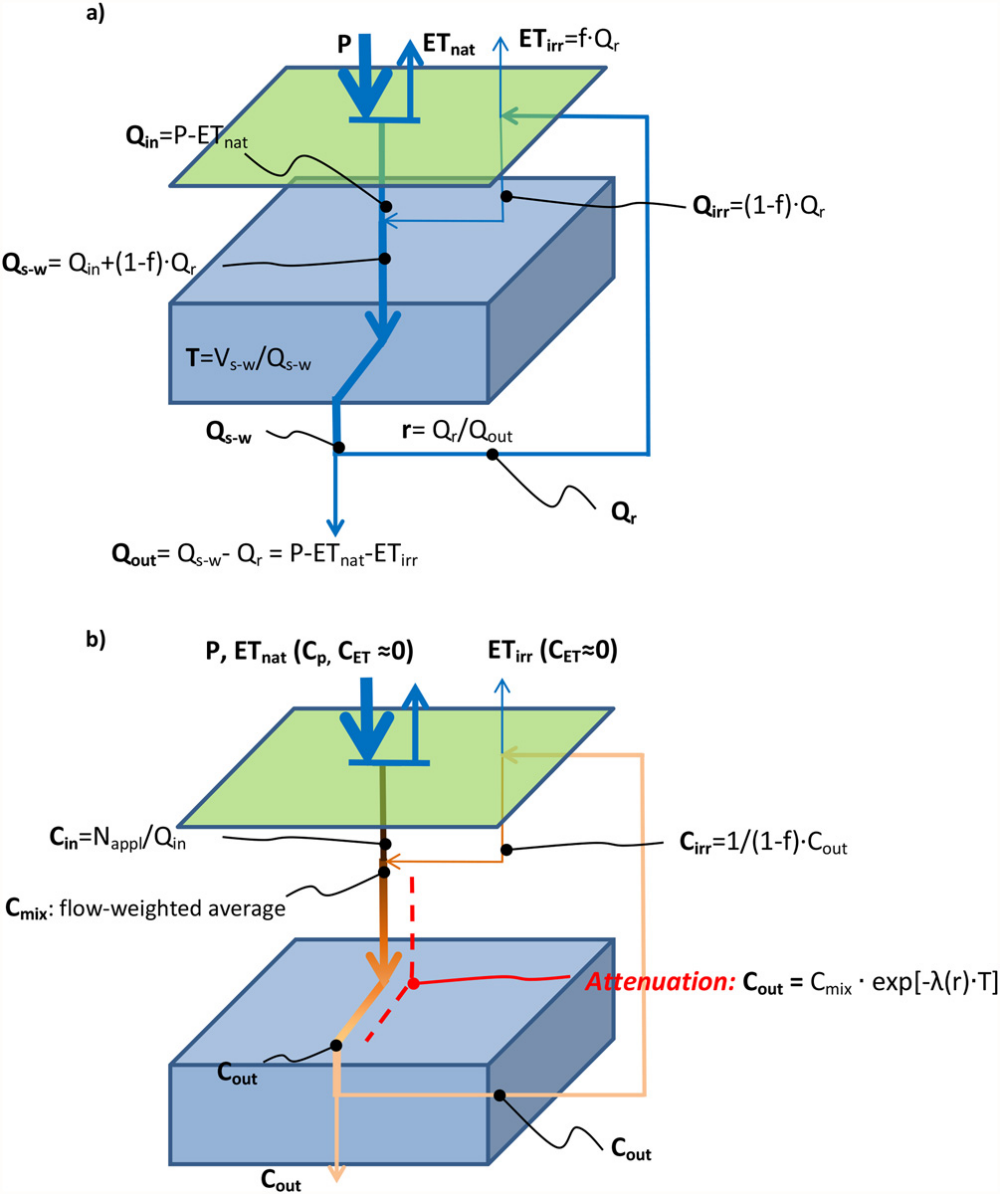
A conceptual model of the impact of basin-scale recirculation of irrigation water on a) water flows and b) N concentrations, where P is precipitation, ETnat is actual ET from precipitation, Qin is inflow from P—ETnat, ETirr is actual ET from irrigation water, r is the recirculation ratio, Qr is recirculated water, f is the fraction of the recirculated water lost through ETirr, Qirr is the fraction of the recirculated water not lost through ETirr, Qs-w is flow through the soil-water system, Qout is the outflow, T is the travel time in the soil-water system, Vs-w is the volume of the soil-water system, Cin is the N concentration in Qin, Cout is the concentration in river water at the outlet, Cirr is the concentration of recirculated water after a certain fraction of the recirculated water has been lost through ET and Cmix is the flow-weighted average of Cin and Cirr. Thickness of the arrows indicates the magnitude of water flows in a specific part of the system.

Flows and flow-paths of the distributed Polflow model were used in creating a lumped conceptual model of the main water flows in ADRB, under the conditions of irrigation (Fig. 2a). On the basis of this conceptual model, the basin-scale, annual average flow, ET and mean advective (water) travel time (T) dynamics was quantified analytically as function of the recirculation ratio (r), defined as the the sum of all water diversions along the river (Q_r_) and the river outflow from the basin (Q_out_) (see the basic relations in Fig. 2). The quantification is based on the assumption that a constant fraction (f) of Q_r_ is lost through ET when it is spread over the irrigated fields, see also previous model assessments from the basin [17; 38; 57]. The f-value was calculated as ET_irr_/Q_r_.

Regarding contaminant transport and transformation along the hydrological transport pathways, we assumed a first-order N attenuation expressed as exp[-λT], where λ is the attenuation rate [T^-1^], and λT [-] is denoted the attenuation product. In order to allow for possible changes in attenuation under different recirculation conditions we let the attenuation rate λ change as a function of r, using a first-order approximation: λ(r) = (1+α·r) λ, where is a constant that allows for different conditions for attenuation from recirculation of water. For instance, positive values of α reflect systems in which the attenuation rate increases with increased recirculation. Values of α between-0.1 and 0.1 reflect systems in which the relation between recirculation and attenuation rate is weak. Systems that can be characterized by positive values of α have previously been observed in laboratory and plot-scale experiments, see e.g., [14; 61–62]. With these assumptions, the output-input concentration ratio C_out_(r)/C_in_ as a function of r can be expressed analytically as (see S1 Text for details and list of variables):

$$C_{out}(r)/C_{in}\;=\;\frac{1+fr}{1+r}\exp\left[\beta \cdot \lambda T\right]/\left(1-\frac{r}{r+1}\exp\left[\beta \cdot \lambda T\right]\right)$$ (1)

where f [-] is the basin-scale fraction of recirculated irrigation water lost through ET and β = (1+α *r*)·((*f*-1)*r*/(1+*fr*)-1)^-1^. Specifically, C_out_ is the concentration in the vicinity of the basin outlet, and C_in_ is the average concentration of upstream runoff (Q_in_ = P-ET_nat_; Fig. 2). For the ADRB an f-value of 0.6, quantified from the Polflow model output (as ET_irr_/Q_r_), was found to be representative for both of the considered periods (1960–1980 and 1981–2000). This value was therefore used in all analytical model predictions of C_out_(r)/C_in_.

We compared if and how the analytical model predictions of C_out_(r)/C_in_ trends, as function of r, could be fitted to observations from ADRB. This comparison was possible due to the considerable irrigation expansion in the ADRB of the past century. Consequently, observational data from the two considered time periods (1960–1980 and 1981–2000) were associated with considerably different r-values. The early period, which represents an early stage of irrigation development and has a low average r-value, was used to determine basin characteristics of α and λT values through calibration. Notably, the α and λT combinations that were found to be consistent with the early period were then maintained unchanged as the model was used to predict C_out_(r)/C_in_ for considerably higher values of r, which represent highly developed irrigation during the late period. A key question was then if the model in its predictive mode with fixed parameter combinations could reproduce observations from the late period too.

Furthermore, in order to express observation data in a way that facilitates comparison with variables of the analytical model, the average C_in_–values (net N input concentration into the basin) of the two observation periods were determined from the average basin-scale N source input [M/T] divided by the corresponding basin-scale Q_in_ [L^3^/T]. The source input was assumed to equal 65% of the N fertilizer application, based on catchment specific field observations of 35% crop uptake of applied N fertilizers [29]. The C_out_-values were taken from the observed average DIN-concentrations measured in the Amu Darya River at the Kziljar station. The actual C_in_-value is associated with uncertainty, because of the existence of other N sources than the documented fertilizer application, such as atmospheric N deposition, waste water and manure. In catchments in Central Valley (California), the share of fertilizer input of the total N input ranges between 25 and 65% depending on agricultural activity in a catchment [63]. Here, uncertainty associated with the lack of basin-specific data on non-fertilizer sources is acknowledged by investigating a relatively wide range of possible conditions, assuming that the ratio between N input from fertilizers and total N input (N_fert_/N_tot_) may range between 25% and 100%. These ranges were used to compare model results with basin estimates on C_in_/C_out_-ratios.

## Results and Discussion

### 3.1 Temporal trends in discharge and DIN concentration

The discharge of the Amu Darya River decreased drastically at the Kziljar gauging station between 1960 and 1980 (blue line and symbols Fig. 1), whereas there was no pronounced discharge trend between 1981 and 2000 due to large inter-annual variability mainly caused by variable weather patterns (red line and symbols Fig. 1). The mean discharge is almost three times as large in the first period 1960–1980 (29 km^3^/year; 95% confidence interval (16; 41); CV = 0.70) than in the later period 1981–2000 (9 km^3^/year; 95% confidence interval (5; 13); CV = 0.84).

The mean P in the Aral region has not changed considerably during the 1960–2000 period and there were no evident trends present (the 10 year running average moves around 257 mm/year; S2A Fig.). However, T has increased by about 1°C during the considered period (based on the 10 year running average; S2B Fig.). In a previous study it has been shown that these climatic changes could not explain the decrease in river flow during this period [17]. Instead, the decrease was shown to be mainly due to expansion of irrigated areas and irrigation water diversions (Table 1; S1a and b Fig.), and related increases of ET losses over the irrigated areas [17; 57; 64]. ET has increased by up to 500 mm over the irrigated areas when comparing pre-1950 conditions to the 1981–2000 period [17]. In more recent years river discharge has been observed to be even lower than in the 1981–2000 period (down to 1 km^3^/year) [65], and future climate change projections show that outlet discharge are likely to totally deplete within a 30-year period [57].

**Fig 3:**
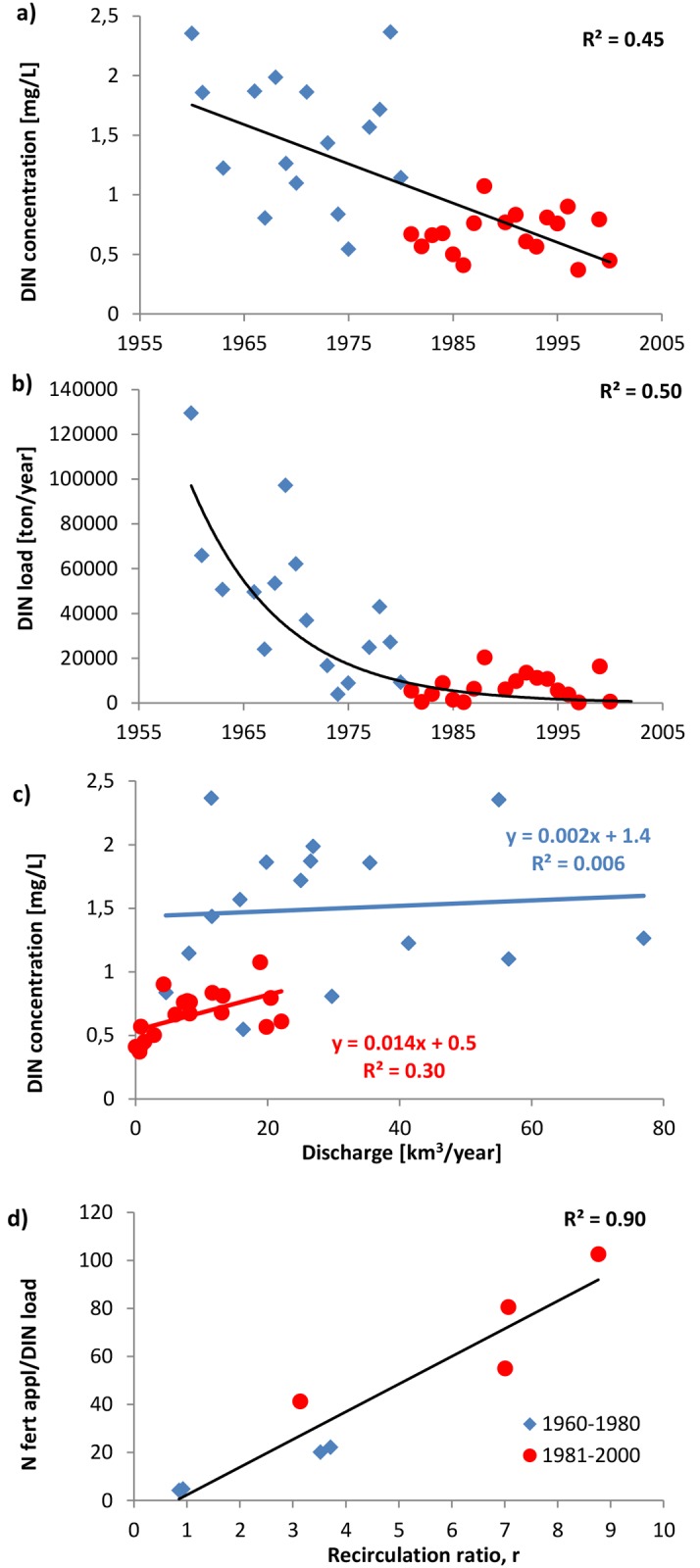
Temporal trend in a) annual mean dissolved inorganic nitrogen (DIN) concentration, and b) annual mean DIN load at Kziljar station, and linear correlation c) between mean annual dissolved inorganic nitrogen (DIN) concentrations and mean annual discharge at Kziljar station for the periods 1960–1980 (blue), 1981–2000 (red) and 1960–2000 (thick black trend line) and d) between five year averages of the ratio of N fertilizer application and DIN load and the recirculation ratio (r) for the Amu Darya River basin for the periods 1960–1980 (blue), and 1981–2000 (red).

During the 40-year period 1960–2000, the DIN concentration of Amu Darya River at Kziljar decreased with time. In the later period 1981–2000 (red circles in Fig.- 3a; mean value 0.68 mg/L; 95% confidence interval (0.57; 0.78); CV = 0.28) the concentration was about half of that in the earlier period 1960–1980 (blue diamonds in Fig. 3a; mean value 1.50 mg/L; 95% confidence interval (1.16; 1.83); CV = 0.36). The composition of DIN was dominated by NO_3_^-^ during both periods (on average 90% of the DIN; S3 Fig.). Similar mean values (1.54 and 0.61 mg/L; 95% confidence interval (1.08; 2.00) and (0.48; 0.75) for the blue and red period, respectively) were obtained when calculating flow-weighted DIN concentrations. Furthermore, alternative estimates of annual average concentrations from monthly data (accounting for intra-annual data gaps, see methods) yielded results that were only 2% higher for the period 1960–1980 and 2% lower for the period 1981–2000. The average monthly concentrations showed relatively similar ranges for the two periods 1960–1980 and 1981–2000, namely 0.01–5.0 mg/L and 0.02–3.4 mg/L, respectively. High concentration events (>1.5 mg/L) were more frequent in the 1960–1980 period than in the 1981–2000 period. The majority of the high concentration measurements were observed in spring and autumn.

The considerable decrease in DIN concentrations with time during 1960–2000, can be shown to be robust with regards to potential impacts of data gaps and identified changes in analysis methods. One of the main reasons is attributed to the changes in NH_4_^+^ concentrations (see the methods section for details), which are low relative to the sum of NO_3_^-^and NO_2_^-^ concentrations. Therefore, they contribute only a small fraction (on average 8%) to estimated total DIN concentrations (S3 Fig.). For instance, if the neglected NH_4_^+^ concentration contributions to DIN (due to missing data 1960–1976) instead were assumed to equal the observed long-term average NH_4_^+^ concentration at the site, it would increase the estimated DIN concentration of the early period (1960–1980) by 8% only. It can also be noted that such a correction slightly enhances the identified trend of decreasing concentrations with time. As further noted in the methods section, the NH_4_^+^ analysis method was changed around 1979; however, the originally used method was employed for such a short time (1977–1979) that it could not considerably bias interpretations of long-term average DIN concentrations (S3 Fig.).

The observed trend of decreasing DIN concentrations in the ADRB is shared with many other large river basins of the world, including large North American rivers like the lower Mississippi River [66] and the Rio Grande River [67], main Japanese rivers [68] and many large European rivers [69]. The decrease in concentrations started in the early 1990s in most of these basins [66–69]. Some of these basins have, like the ADRB, been subject to considerable agricultural development and irrigation expansion, e.g., the Ebro River basin [70] and the lower Mississippi river basin [71]. Urban N sources have changed considerably in many of the basins during the considered time period, and improved wastewater treatment has in particular contributed to observed N concentration decreases [67–69]. Notably, conclusive results on the possible contribution of agricultural changes to observed N concentration decreases, or their importance relative to the urban changes, have as of yet not been reported.

### 3.2 Potential mechanisms behind observed decrease in DIN concentration

#### 3.2.1 Basin scale change in input of N sources

In the ADRB, N fertilizer application rates peaked around 1980. On average, the rates were estimated to be about 20% higher in the later 1981–2000 period than in the earlier 1960–1980 period (Table 1 and 2). Moreover, fertilization efficiency in terms of cotton yield has reportedly decreased to about 50% in Uzbekistan after its independence in 1991 due to inadequate N fertilizer application management [35]. In addition, the agricultural area increased considerably throughout the 40-year period between 1960 and 2000. Since the changes in rainfed agricultural area are negligible, this overall increase is associated with increasing areas of irrigation, from about 2,045,000 ha in 1960 to 3,650,000 ha in 2000 (Table 1). Due to the combined effects of the changes in specific N application and the expanded agricultural areas, the total application of N fertilizer increased nearly threefold between 1960 and 1980, and was sustained at a high level between 1980 and 2000 (Table 1 and 2; S1C Fig.). The DIN concentrations have thus decreased despite an increase throughout the 40-year period, and cannot be related to the documented changes in the application of N fertilizers in the catchment.

**Table 2:** Mean values of fertilizer application in kgN/ha and ton/year, crop yield in ton/ha, N uptake per area in kgN/ha, N content in crops in kgN/ton, total N uptake by crops in ton/year and net N input in ton/year (NNI; N fertilizer application minus N uptake) for cotton and wheat fields in the Uzbek part of the Amu Darya river basin (UADRB) for the periods 1960–1980 and 1981–2000.

Period	N fertilizer application rate in UADRB [kgN/ha]	Total N fertilizer application in UADRB [ton/year]	Crop yield in UADRB [ton/ha]	N uptake per ha cultivated area in UADRB [kgN/ha]	N content in crops in UADRB [kgN/ton]	Total N uptake by crops in UADRB [ton/year]	Net N input in UADRB [ton/year]
**1960–1980**	160	194,000	3.4	144	42.3	171,500	22,500
**1981–2000**	197	283,000	3.7	137	37.0	199,000	84,000

Other DIN inputs to the basin’s water system could come from, e.g., wastewater, livestock production and atmospheric deposition. There is lack of data on atmospheric N deposition in the ADRB. In regions at the same latitude as ADRB; however, atmospheric N deposition has only contributed to the total DIN input by about 13% (for the year 1990) [72]. Due to this relatively low contribution compared to N fertilizer input, which is about six times larger than atmospheric N deposition in these regions [72], we believe that changes in atmospheric deposition would not have a large impact on the basin scale DIN concentration trends. Further, livestock production (in terms of number of animals) decreased by on average 57% in Kyrgyzstan, Uzbekistan, Tajikistan and Turkmenistan after the Soviet Union collapse (data from the FAO STAT database). However, the collapse happened many years after the downward shift of riverine concentrations, and could hence not be the cause of it.

There is a lack of direct data and information on trends in DIN input to the basin from wastewater. However, sewage treatment systems in the basin are generally reported to be more than 30 years old and badly maintained, e.g., [73]. There are large differences in access to sewage systems between urban and rural areas. In Uzbekistan, 46% of the urban population and only 0.3% of the rural population (which constitutes about 65% of the total population) are reported to be connected to a sewage system [74]. In addition, the population of the basin has increased by about 65% between the 1960–1980 and the 1981–2000 periods [75], which contributes to higher pressures on the existing sewage systems in the later period. In regions at the same latitude as ADRB, sewage has only contributed to the total DIN input by about 10% (year 1990) [72]. Taken together, this provides a sound basis for believing that sewage system development cannot have significantly contributed to basin-scale N load decreases.

#### 3.2.2 Basin scale change in N crop uptake

Riverine N concentrations may however also be influenced by changes in N uptake by crops, which is caused by changes in crop types or crop yield. Increase in N uptake leads to increase in removal of N from the system and can therefore in principle explain decreased concentrations in downstream water systems. Wheat and cotton are the two major crops cultivated in the catchment. According to a field study from Uzbekistan, the cotton uptake of N (160 kgN/ha) is about 60% higher than the wheat uptake per hectare (99 kgN/ha) at commonly used N fertilizer rates [29]. Wheat cultivation has increased in the catchment at the expense of cotton after the independence in 1991 [33; 35]. Cotton yields have increased from 2.4 ton/ha in 1960 to 4.5 ton/ha in 1980. Thereafter, the yields have decreased to around 2.8 ton/ha in 2000 [35]. Assuming a similar pattern for all crop types, the difference in average yield of the earlier (1960–1980) and the later (1981–2000) periods is hence on the order of 10% (Table 2).

The N uptake per hectare cultivated area in the Uzbek part of the basin, for which refined data of crop uptake and yield was available [29], was slightly lower in the later period than in the earlier period due to changes in crops. Also the N content in crops (kg N/ton) was lower in the later period (Table 2). However, the total N uptake in crops (ton/year) has increased due to the agricultural expansion (Table 2). Since the total fertilizer application has increased much more (+ 46%; Table 2) than the total N uptake by crops (+16%; Table 2), the net N input (NNI; the difference between N application and N uptake by crops) has also increased. Such increased NNI into the system can of course not explain the observed decrease in riverine N concentrations after 1980, since it should act to increase rather than decrease the concentrations.

#### 3.2.3 Basin scale change in irrigation efficiency

The water diversions in cubic meter per hectare irrigated area have increased by about 30% between 1960 and 1980 and decreased by 35% between 1981 and 2000 in the ADRB (Table 1). Improved irrigation efficiency (here defined as the ratio between water used by the crop and water diverted for irrigation) during the later period allows for smaller water diversions per hectare, which may change leakage of N and resulting DIN concentrations. Even though this could potentially decrease DIN concentrations, no studies have been found to support this effect. Rather, for example in the semi-arid Ebro River basin in Spain, irrigated areas with high irrigation efficiency were associated with higher NO_3_^-^ concentrations in the return flows compared to areas with low irrigation efficiency. This was explained by higher evapo-concentrations of irrigation water in the root zone when irrigation efficiency was increased [76]. This results in higher DIN concentrations due to smaller amounts of irrigation water to dilute the leaching N from the fields. Another study from the same catchment further showed that observed river water concentrations of N increased, even though N fertilization decreased, when the irrigation efficiency was improved [77]. It is thereby plausible that improved irrigation efficiency in the ADRB may have increased rather than decreased riverine DIN concentrations.

#### 3.2.4 Local scale changes due to river reservoirs

Local dam and reservoir constructions in the Amu Darya River could also influence DIN concentrations, through altered attenuation of N rich upstream water during reservoir storage, see e.g., [20]. Several dam constructions were new at the time of the late period, including the Tuyamuyn reservoir [36]. This provides a potential explanation for the lower DIN concentrations observed at that time period. Ratios of observed concentrations between the early period (1960–1980) and the late period (1981–2000) were estimated along the Amu Darya River to evaluate the influence of such local impoundments (Table 3; Fig. 1). Apparent from these results is that DIN concentration ratios were consistently around 0.5, regardless of river location. If reservoirs constructed along the river in the late period had major impact on the N removal, this would have been reflected in decreasing ratios from the most upstream river location (Termez) to the following downstream river locations, with N being increasingly removed between each location. Even though local impoundments like constructed dams and reservoirs are known to impact N concentrations [20], the spatially consistent reduction in DIN concentrations by 50% in the late period cannot be attributed solely to specific local effects in the river. The mechanism behind these spatially consistent decreasing concentrations rather point to processes occurring throughout the basin.

**Table 3:** Average DIN concentrations and DIN concentration ratios based on average DIN concentrations for the two periods 1960–1980 and 1981–2000 at four locations within the Amu Darya River Basin (ADRB).

Station	Distance (km) upsteam of Kziljar	DIN concentration 1960–1980 [mg/L]	DIN concentration 1981–2000 [mg/L]	DIN concentration ratio between early and late period
**Termez**	1095	1.73	0.86	0.49
**Tuyamuyn**	340	1.36	0.75	0.55
**Nukus/Chatly**	110	1.50	0.72	0.48
**Kziljar**	0	1.50	0.65	0.44

#### 3.2.5 Basin scale changes in recirculation of irrigation water

**Fig 4:**
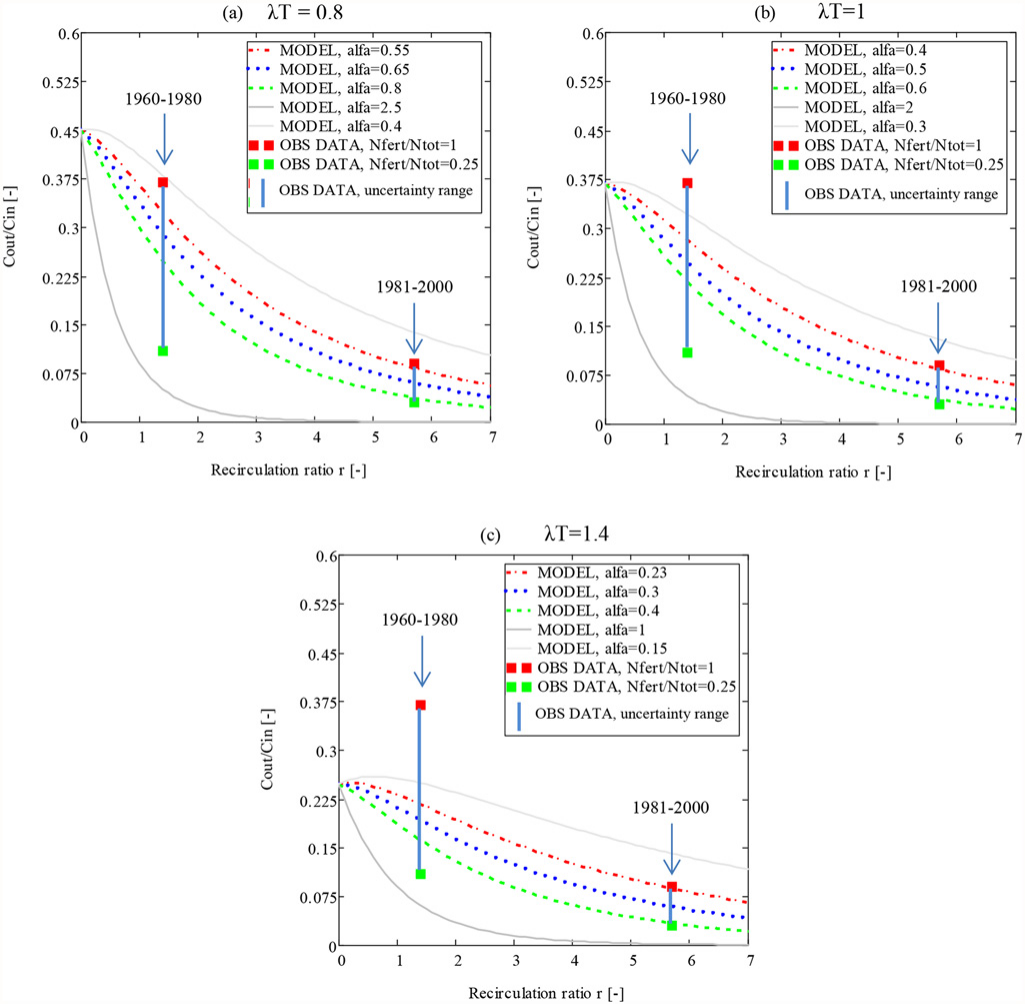
Concentration ratios (Cout(r)/Cin), calculated with Eq 1 in section 2.3, for different recirculation ratios (r), attenuation products (λT) and attenuation constants (α). Cout(r)/Cin is shown for: a) λT = 0.8; α = 0.4, 0.55, 0.65, 0.8 and 2.5, b) λT = 1; α = 0.3, 0.4, 0.5, 0.6 and 2 and c) λT = 1.4; α = 0.15, 0.23, 0.3, 0.4 and 1. Cout/Cin uncertainty intervals for the two time periods, assuming Nfert/Ntot ranging between 0.25 and 1, are indicated in blue solid lines at r for respective period. r for periods 1960–1980 and 1981–2000 equaled 1 and 6, respectively.

The basin scale development of irrigation canal-drainage-reservoir systems has gradually expanded (S1a and b Fig.) while the discharge has decreased (Fig. 1) during the considered 40-year period. This expansion is reflected in the recirculation ratio (r = Q_r_/Q_out_) of irrigation water, with Q_r_ becoming larger than Q_out_ at Kziljar after 1970, with an average r-value of 1.4 for the 1960–1980 period. With continued irrigation expansion and decreased discharge, the r-value doubled during the early 1980s and resulted in an average r-value of 5.7 for the full 1981–2000 period. These changes in r can be compared with changes in the basin-scale C_out_/C_in_ ratio. Our quantification (section 2.3) yielded an observation-based C_out_/C_in_ ratio of ADRB between 0.11 and 0.37 in the period 1960–1980, which decreased to between 0.03 and 0.11 in the period 1981–2000 (Fig. 4; vertical blue lines). The low concentration ratios of the latter period reflect high N attenuation of approximately 99%, which can be compared with the reported attenuation (92%) of intensely irrigated sub-catchments in the Ebro River basin in Spain [16].

Further, Fig. 4 illustrates three different sets of hydrological parameters, namely (a) λT = 0.8; 0.6≤α≤0.8, (b) λT = 1; 0.4≤α≤0.6, and (c) λT = 1.4; 0.2≤α≤0.4, for which the model results of equation (1) (C_out_/C_in_; colored curves) fell within the C_out_/C_in_-range derived from ADRB observations (vertical blue line between filled squares). From the early (1960–1980) period match, shown in the left part of the Fig. 4 panels, we infer that the considered parameter combinations of (a) to (c) (i.e., roughly 0.8≤λT≤1.4; 0.2≤α≤0.8) reflect and delimit basin characteristics under low degrees of irrigation water diversion and recirculation (r = 1.4). With each of the curves of Fig. 4 representing fixed parameter values, the right part of the Fig. 4 panels shows that the model could reproduce observations also from the highly irrigated late period 1981–2000 (r = 5.7) without need of recalibration. This illustrates that the proposed model can have predictive capabilities. For the sake of completeness, the grey curves of Fig. 4 show example parameter combinations of the analytical relation (1) that did not yield a match with observation data. Notably, for negligible to relatively weak attenuation effect of recirculation (0≤ α<0.2), the analytical curve (1) could not be matched with observations, implying that this attenuation effect must be considerable at the basin scale.

These quantified basin-scale relations between changed N attenuation and increasing recirculation of irrigation water have previously only been observed in laboratory and plot-scale experiments [14; 61–62]. For instance, in well-controlled sand filter systems with recirculation, N attenuation has been observed to increase from 45% to 70% as r increases from 1 to 3 [61]. Furthermore, in paddy fields of the Shiga Prefecture in Japan, N attenuation increased from 43% to 66% as r increased from 1 to 4 [62]. In addition, a high correlation between increased r and decreased N concentration ratios between outflow and inflow was observed in the Yoshinuma region of Japan [14]. However, only r values much lower than 1 were considered. The observed basin-scale increase in attenuation in this study from about 85% (r = 1.4) to about 99% (r = 5.7) is hence of similar significance in terms of magnitude of change as reported in [61] and [62].

Although relations between r and N attenuation have not been quantified in large drainage basins before, it has for instance been seen that enhanced N attenuation of sub-catchments of the semi-arid Ebro River, Spain, is related to high percentage of sub-catchment area drained into irrigation reservoirs [16]. Furthermore, recirculation of irrigation water was identified as a mechanism that could contribute to the relation [16]. Similarly, a significant negative least-square coefficient between irrigated area and delivery of N from land to water has been found in the Missouri River basin [78]. The negative relationship implies that the denitrification favored by irrigation outweighed N leaching from agriculture at the basin scale [78]. In agreement, agriculture has also been identified as an N sink in the Middle Rio Grande, due to processes such as direct uptake by crops and denitrification [79].

In general, the range of attenuation product values λT for N obtained from the present curve fitting is consistent with independently reported field-scale assessments (with ranges of 1≤ λT≤10) [80–81]. The range of reported λT for N reflects varying hydrologic, transport and attenuation conditions of the investigated catchments, such as flow-pathways, geochemical conditions along the pathways and T distributions. The ADRB characteristics do not differ considerably from the range of conditions considered in [80] and [81], which can contribute to the above reported consistency in λT. For instance, travel times from the irrigated fields of the ADRB to the basin outlet are generally within the range of 0.7 to 6 years that was considered in [81].

The here quantified ADRB relations between r and DIN attenuation, together with these observations at agricultural plots [14; 62], and agriculturally dominated catchments [16; 78–79], raises the question of to which extent irrigation-related changes can have contributed to observed DIN decreases in large catchments of mixed urban-rural sources in many regions of the world, in addition to already identified impacts of improved wastewater treatment [66–69].

### 3.3 Temporal trends in DIN load and expected future change

The DIN load of the Amu Darya River at the downstream Kziljar gauging station was on average approximately 6 times lower in the later period 1981–2000 (red; mean value 6,900 ton/year; 95% confidence interval (3,500; 10,300); CV = 0.89) compared to the earlier period 1960–1980 (blue; mean value 43,900 ton/year; 95% confidence interval (23,000; 64,900); CV = 0.77; Fig. 3b). The decrease in load was the combined result of both the drastically reduced discharge and the decreased concentrations. The presented annual loads of DIN were calculated as the product of mean annual concentration and mean annual water discharge. Refined calculations using the sum of the monthly concentrations times monthly discharge (where missing months were estimated from concentration and discharge pattern of an upstream station) gave loads that were on average 1% lower for the period 1960–1980 and 14% higher for the period 1981–2000. This deviation is small relative to the observed 600% difference in loads between the periods (Fig. 3b).

The correlation between inter-annual discharges and loads was high for the whole 40-year period (S4 Fig.), which is related to the fact that load equals discharge times concentration. However, discharge and concentration did not show any correlation (1960–1980) or only weak positive correlation (1981–2000; Fig. 3c), indicating that increasing/decreasing discharge was commonly not accompanied by similar increases/decreases in concentrations. This means that the lower loads in the later period are predominantly, but not exclusively, connected to the lower discharges during that period (also shown by the high correlation in S4 Fig.). These results are consistent with those of [21], who showed a pattern of inter-annual variation in N load that was highly dependent on discharge in the intensively managed Mississippi Atchafalaya River basin and the Baltic Sea drainage basin. Such relationships can therefore be useful in predicting nutrient loads from discharge data. The present ADRB results additionally showed a statistically significant difference between the linear regression coefficient for N loads and discharges for the two considered 20-year periods (S4 Fig.). According to the present results, this shift was due to increased DIN attenuation in the later period compared to the earlier period caused by increased irrigation and recirculation of water in the irrigation-drainage-reservoir system. This shows that, on decadal scales, the basic hydrological functioning of large basins, and specifically the dynamics between discharge and load, can shift in response to changes in management practices.

Using the same definitions as Thompson et al. [82], who studied N loading in 31 catchments in the US and Puerto Rico, the ADRB has changed from a high export basin during the 1960–1980 period (having a normalized mean annual DIN load of 0.49, defined as the mean annual load per catchment area (g m^–2^ yr^–1^) divided by the largest mean annual load of the considered American catchments) to a low export basin during the 1981–2000 period (having a normalized mean annual total DIN load of 0.08). Furthermore, the ratios between the N fertilizer application and N river outlet load were of the same order of magnitude in the ADRB (around 80 for the second period; Fig. 3d) as in many other arid and semi-arid river basins such as the Murray-Darling, the Orange and the Zambezi (29–94) [83]. The Nile drainage basin has a six times greater ratio of 490 [83]. Overall, these ratios are relatively high, which can be explained by generally favorable conditions for N attenuation processes in the terrestrial landscape in arid and semi-arid river basins, small river flows, and partly by the presence of large river reservoirs in these regions [20; 83–84]. However, in the ADRB, dam constructions were shown to have negligible effects for DIN concentration decreases (see section 3.2.4). For the whole period (1960–2000), high linear correlations between 5-years averages of r and N fertilizer application-DIN river outlet load ratios were found for the ADRB (R^2^ = 0.90; Fig. 3d). This is in agreement with the identified relationship between N attenuation and r from the analytical modeling.

The Ebro and Danube rivers of Europe are, like the ADRB case, associated with decreasing N loads both due to decreasing discharge and N concentrations [12]. However, in contrast to the ADRB case, a considerable N load decrease of the Yellow River is solely due to a large decrease in discharge [85]. More generally, such changes in discharge can impact N export from many of the world’s river basins. For example, the four largest rivers in Africa (Zambezi, Niger, Nile and Zaire) are projected to show decreased N loads for the period 2000–2050 due to decreased discharge mainly caused by climatic changes and human consumption increases [23]. Globally, considerable changes in recirculation are primarily expected in arid and semi-arid regions, which are generally characterized by low river discharge and high dependence on irrigation for agriculture. For instance, the projected global average increase in irrigation water withdrawals from 2005 to 2050 is 11% [86]. Due to high water losses, river discharges would then decrease correspondingly in many arid and semi-arid regions. Assuming similar conditions of N attenuation as in the present study (i.e., the same values on r, β, f and λ as in equation 1), this would lead to an increase in r from just below 6 to more than 7 in arid and semi-arid regions, implying regionally increased DIN removal and decreased DIN export by as much as 40%. The current DIN export from arid and semi-arid regions constitutes about 4% of the global DIN export, or in absolute terms about 1 Tg/year [87–88]. This implies a potential future decrease in DIN export from arid and semi-arid regions of about 0.4 Tg/year, as a result of expected increases in water recirculation.

As mentioned earlier, the reduced Amu Darya river discharge has thus far been driven by the increased irrigation water withdrawals and not by climatic changes [17]. Hence, the climate change contribution to observed changes in total flows, travel times and attenuation processes has likely been small. However, parallel research shows that projected future climate change may cause reductions in river flow [57]. With maintained or increased water extraction for irrigation, this means that the recirculation ratio would increase, since a larger part of the reduced water discharges then would be recirculated. This could hence contribute to further decreases of DIN concentrations and loads.

Further, the quantification of relations developed here might also be relevant for basin scale predictions of other pollutants than DIN. Recirculation of irrigation water on small scales has for example been shown to also reduce phosphorous [19; 89], suspended sediment and organic matter concentrations and loads [62]. Combining these plot scale results with the here developed quantifications suggests that, for example, metals commonly associated with suspended sediments, e.g., [90], and organic matter, e.g., [91], may undergo similar concentration and load reductions through cyclic irrigation.

## Conclusions

Decreasing riverine DIN concentrations of about 50% were observed in the ADRB near its Aral Sea outlet during a 40-year period of intensified irrigated agriculture, despite increased N fertilizer application of 20% and increased net N input (NNI). The observed concentration trend is hence more related to changed hydrological conditions within the catchment than changed external mass input.

Results show that the decreased DIN concentrations were primarily due to increased recirculation of irrigation water causing increased flow-path lengths with associated enhanced N attenuation.

An analytic expression between the recirculation ratio (defined as the basin-scale irrigation water withdrawals divided by the outflow) and C_out_/C_in-_ratios was developed. The resulting curve, estimating effective attenuation effects of increased recirculation, could be fitted to basin-scale observations of ADRB. The results imply that the increase in basin-scale attenuation processes due to recirculation can be considerable, as previously seen at smaller scales, in well-controlled laboratory experiments and at agricultural plots.

Furthermore, increased recirculation can have contributed to observed increases in N attenuation in other agriculturally dominated drainage basins subject to irrigation expansion, in different parts of the world. Regarding N concentration reductions in catchments of mixed urban-rural N sources, the results imply that effects of changes in irrigation relative to effects of changes in wastewater treatment may need further attention.

The observed six-fold decrease in DIN export from the ADRB during the considered 40-year period was a combined result of drastic river flow reduction and decreased DIN concentrations at the basin outlet, both of which were shown to be caused by agricultural development.

Several arid and semi-arid regions around the world are projected to undergo similar reduction in discharge as in the ADRB due to climate change and agricultural intensification, and may therefore undergo comparable shifts in DIN export and storage as already observed in the ADRB. Further, the methodological approach developed here may be relevant for assessing basin scale remediation of pollutant beyond N, including for example metals and organic matter.

## Supporting Information

S1 Fig Five years mean of a) irrigated area [39–40], b) irrigation water diversion [39] and c) total N-fertilizer application, data from FAO’s statistical database FAOSTAT (available at faostat.fao.org) and [35], in the Amu Darya river basin. (TIF)

S2 Fig Mean annual a) temperatures and b) precipitation in the Aral region, where solid lines show the 10 year running averages. (TIF)

S3 Fig Concentrations of nitrate (NO_3^-^_), nitrite (NO_2^-^_), ammonium (NH_4^+^_) and DIN (sum of NO_3^-^_. NO_2^-^_ and NH_4^+^_) shown in logarithmic scale. (TIF)

S4 Fig Linear correlation between mean annual dissolved inorganic nitrogen (DIN) loads and mean annual discharge at Kziljar station for the periods 1960–1980 (blue), 1981–2000 (red) and 1960–2000 (thick black trend line). (TIF)

S1 Text Supporting text. (PDF)
