# Supplementary figures and images for: Mechanisms of Basin-Scale Nitrogen Load Reductions under Intensified Irrigated Agriculture
Source: PLoS One. 2015 Mar 19;10(3):e0120015. doi: 10.1371/journal.pone.0120015 (PMC4366109; doi:10.1371/journal.pone.0120015)

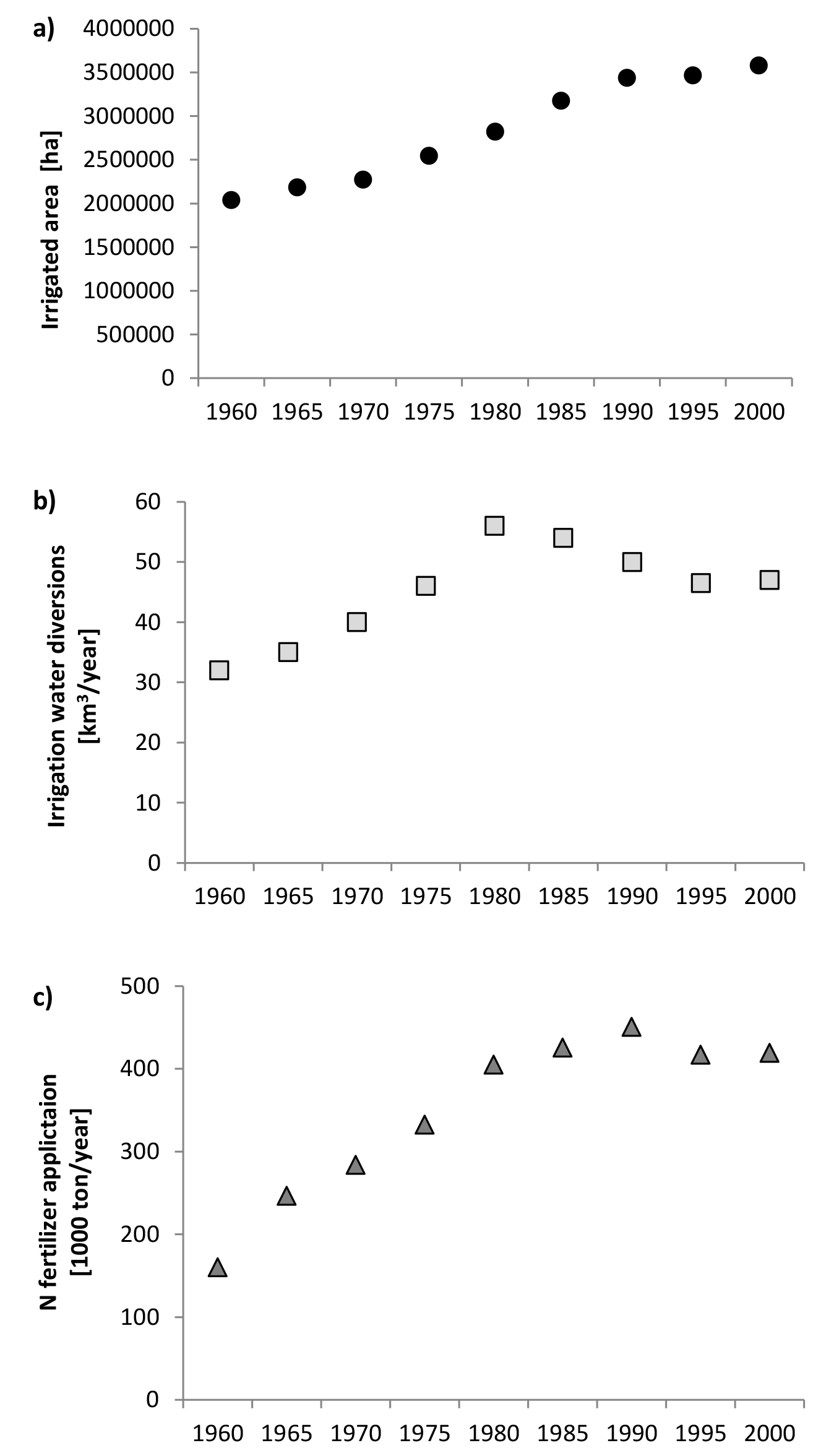

Supplement: S1 Fig — (TIF) [file pone.0120015.s001.tif]

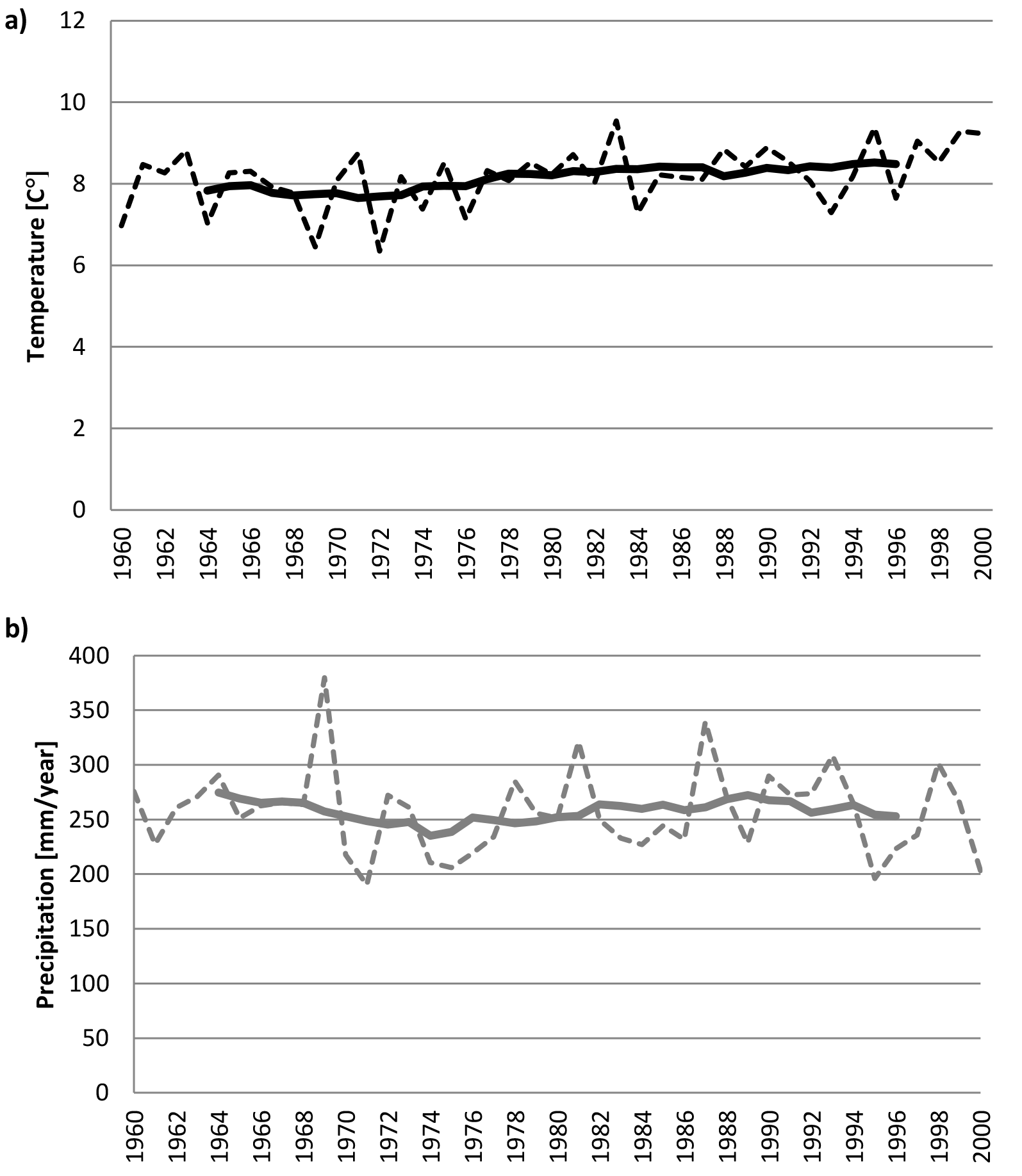

Supplement: S2 Fig — (TIF) [file pone.0120015.s002.tif]

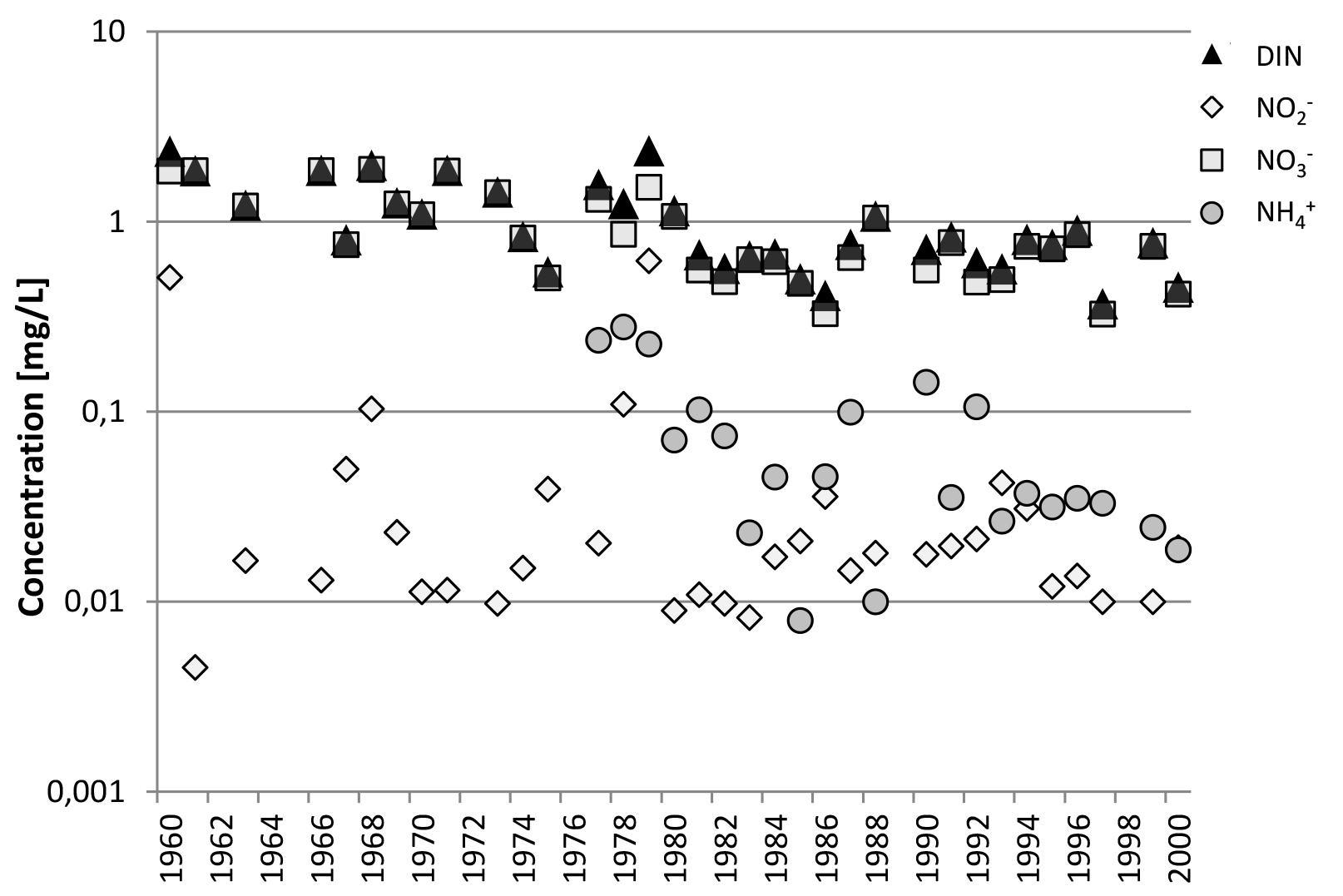

Supplement: S3 Fig — (TIF) [file pone.0120015.s003.tif]

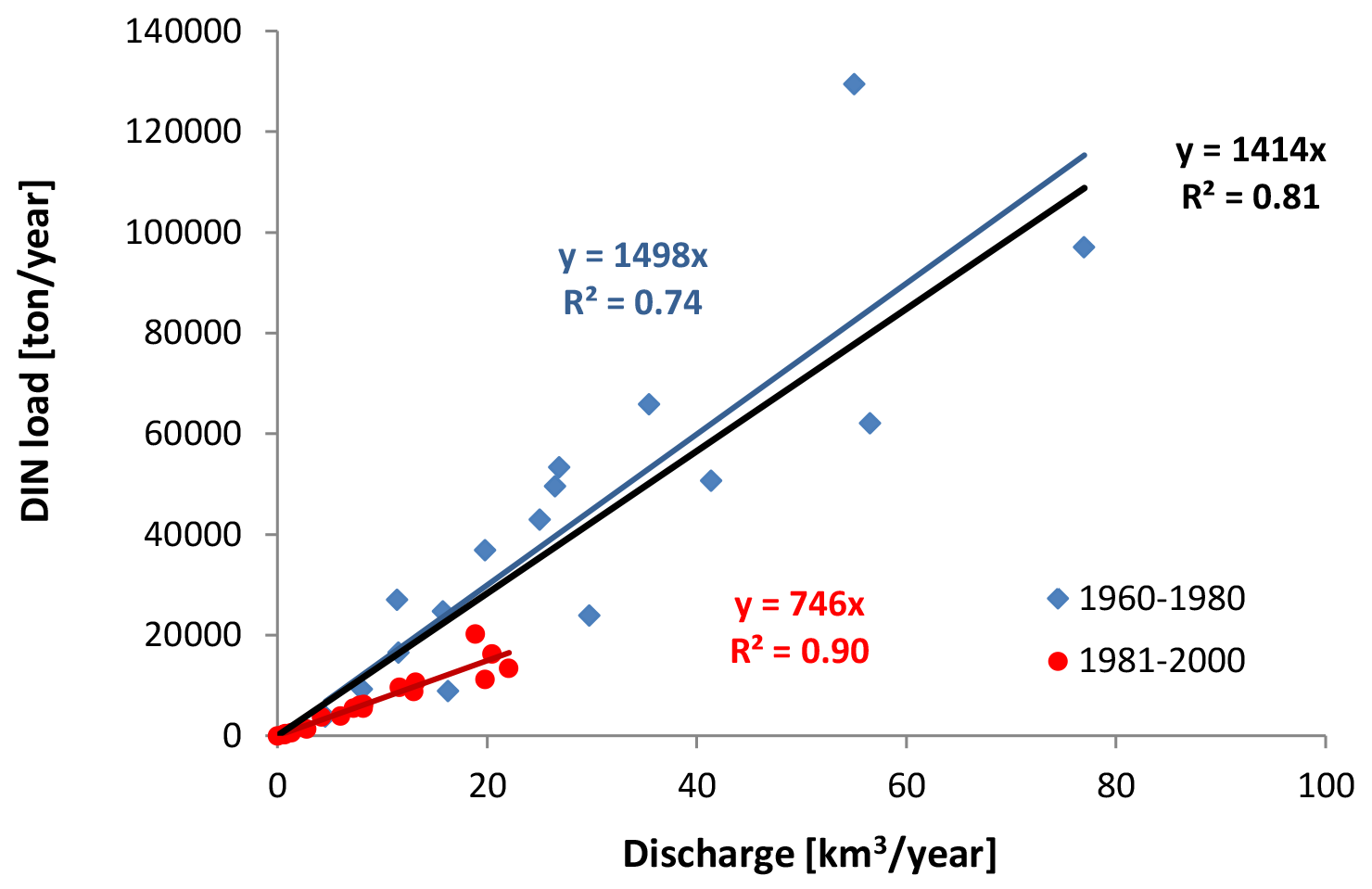

Supplement: S4 Fig — (TIF) [file pone.0120015.s004.tif]
