# Supplementary material for: Mechanisms of Basin-Scale Nitrogen Load Reductions under Intensified Irrigated Agriculture
Source: PLoS One. 2015 Mar 19;10(3):e0120015. doi: 10.1371/journal.pone.0120015 (PMC4366109; doi:10.1371/journal.pone.0120015)
Supplement: S1 Text — (PDF) [file pone.0120015.s005.pdf]

SUPPLEMANTARY INFORMATION

Mechanisms of basin-scale nitrogen load reductions under intensified irrigated agriculture

Rebecka Törnqvist, Jerker Jarsjö, Josefin Thorslund, P. Suresh C. Rao, Nandita B. Basu and Georgia Destouni

---

Derivation of the relation between the basin-scale ratio of average nutrient concentration C_{out} at the outlet of the basin and concentration C_{in} in the runoff, and recirculation ratio, r (Equation 1 of the main paper).

Flow scheme and notation are according to Figure 2 of the main paper.

Determine the relation between Q_{in}, Q_{out}, and r.
From figure 2: $Q_{out}=Q_{in}-ET_{irr}=Q_{in}-f\cdot Q_r$, where $Q_r=r\cdot Q_{out}$ per definition. Hence,

$$Q_{in}/Q_{out}=1+f\cdot r \qquad (1)$$

Determine C_{mix} from flow-weighted averaging of C_{in} and C_{irr}

$$C_{mix} = \frac{C_{in}\cdot Q_{in}+C_{irr}\cdot Q_{irr}}{Q_{in}+Q_{irr}} \qquad (2)$$

Express C_{mix} as function of C_{in}, C_{out}, f, and r. Insert $C_{irr}=1/(1-f)\cdot C_{out}$ and $Q_{irr}=(1-f)\cdot Q_r$ (Figure 2) into (2). Divide the numerator and the denominator of (2) with Q_{out} and insert Equation (1). This yields:

$$C_{mix} = \frac{C_{in}(1+f\cdot r)+C_{out}\cdot r}{1+r} \qquad (3)$$

Assume that N undergoes first-order attenuation in the soil water-groundwater system (blue box of Figure 2). For first order decay we then have per definition:

$$C_{out} = C_{mix}\cdot \exp[-\lambda(r)\cdot T(r,f)] \qquad (4)$$

in which $T(r,f)$ equals

$$T(r,f) = V_{s-w}/Q_{s-w}(r,f) \qquad (5)$$

where V_{s-w} and $Q_{s-w}(r,f)$ are the water-filled volume of, and water flow through the soil-water system. Inserting $Q_{s-w}(r,f)= Q_{in}+(1-f)Q_r$ (see Figure 2), $Q_r=r\cdot Q_{out}$ (per definition) and $Q_{out}=Q_{in}/(1+f\cdot r)$ (from (1)) into (5) yields

$$T(r,f) = \frac{V_{s-w}}{Q_{in}} \cdot \left(1 + \frac{(1-f)\cdot r}{1+f\cdot r}\right)^{-1} = T_{r=0} \cdot \left(1 + \frac{(1-f)\cdot r}{1+f\cdot r}\right)^{-1} \quad (6)$$

Inserting (6) into (4), expressing the degradation rate λ as a function of r according to $\lambda(r)=(1+\alpha\cdot r)\lambda$ (section 2.3 of the main text), and letting $T=T_{r=0}$ denote the mean travel time under conditions of no recirculation, we obtain:

$$C_{out} = C_{mix} \cdot \exp\left[-\lambda T(1+\alpha r) \cdot \left(1 + \frac{(1-f)\cdot r}{1+f\cdot r}\right)^{-1}\right] \quad (7)$$

Inserting (3) into (7), solving the resulting equation for C_{out}, and dividing by $C_{in,}$ we obtain Equation (1) of the main paper.

List of variables

α – Attenuation constant

λ – Attenuation rate

λT – Attenuation product

C_{out}/C_{in} - Output-input concentration ratio

C_{in} – Concentration of upstream river runoff

C_{irr} - Concentration of recirculated water

C_{mix} - Flow-weighted average of C_{in} and C_{irr}

C_{out} - Concentration in outlet river runoff

ET_{irr} - Evapotranspiration from irrigation water

ET_{nat} – Natural evapotranspiration without irrigation

f – Fraction of irrigation water lost through evapotranspiration

Q_{irr} – Fraction of the recirculated water not lost through ET_{irr}

Q_{in} – Upstream river discharge

Q_{out} – Outlet river discharge

Q_{s-w} – Total discharge in soil-water system

Q_r – Recirculated water

r – Recirculation ratio

T - Advective travel time

V_{s-w} – Volume of soil-water system
